# Outcome reporting in randomised controlled trials and meta-analyses of appendicitis treatments in children: a systematic review

**DOI:** 10.1186/s13063-015-0783-1

**Published:** 2015-06-17

**Authors:** Nigel J. Hall, Mufiza Z. Kapadia, Simon Eaton, Winnie W. Y. Chan, Cheri Nickel, Agostino Pierro, Martin Offringa

**Affiliations:** Faculty of Medicine, University of Southampton, Southampton, UK; Department of Paediatric Surgery and Urology, Southampton Children’s Hospital, Southampton, UK; Toronto Outcomes Research in Child Health (TORCH), SickKids Research Institute, Toronto, Canada; Developmental Biology Programme, UCL Institute of Child Health, London, UK; Hospital Library and Archives, The Hospital for Sick Children, Toronto, Canada; Division of General and Thoracic Surgery, The Hospital for Sick Children, Toronto, Canada

**Keywords:** appendicitis, children, outcome measures, core outcome set, patient-reported outcome measures, systematic review, surgery

## Abstract

**Background:**

Acute appendicitis is the most common surgical emergency in children. Despite this, there is no core outcome set (COS) described for randomised controlled trials (RCTs) in children with appendicitis and hence no consensus regarding outcome selection, definition and reporting. We aimed to identify outcomes currently reported in studies of paediatric appendicitis.

**Methods:**

Using a defined, sensitive search strategy, we identified RCTs and systematic reviews (SRs) of treatment interventions in children with appendicitis. Included studies were all in English and investigated the effect of one or more treatment interventions in children with acute appendicitis or undergoing appendicectomy for presumed acute appendicitis. Studies were reviewed and data extracted by two reviewers. Primary (if defined) and all other outcomes were recorded and assigned to the core areas ‘Death’, ‘Pathophysiological Manifestations’, ‘Life Impact’, ‘Resource Use’ and ‘Adverse Events’, using OMERACT Filter 2.0.

**Results:**

A total of 63 studies met the inclusion criteria reporting outcomes from 51 RCTs and nine SRs. Only 25 RCTs and four SRs defined a primary outcome. A total of 115 unique and different outcomes were identified. RCTs reported a median of nine outcomes each (range 1 to 14). The most frequently reported outcomes were wound infection (43 RCTs, nine SRs), intra-peritoneal abscess (41 RCTs, seven SRs) and length of stay (35 RCTs, six SRs) yet all three were reported in just 25 RCTs and five SRs. Common outcomes had multiple different definitions or were frequently not defined. Although outcomes were reported within all core areas, just one RCT and no SR reported outcomes for all core areas. Outcomes assigned to the ‘Death’ and ‘Life Impact’ core areas were reported least frequently (in six and 15 RCTs respectively).

**Conclusions:**

There is a wide heterogeneity in the selection and definition of outcomes in paediatric appendicitis, and little overlap in outcomes used across studies. A paucity of studies report patient relevant outcomes within the ‘Life Impact’ core area. These factors preclude meaningful evidence synthesis, and pose challenges to designing prospective clinical trials and cohort studies. The development of a COS for paediatric appendicitis is warranted.

**Electronic supplementary material:**

The online version of this article (doi:10.1186/s13063-015-0783-1) contains supplementary material, which is available to authorized users.

## Background

Acute appendicitis is the most common surgical emergency in children [1]. The lifetime risk of developing appendicitis is 7-8 % with a peak incidence in the early teenage years. Traditionally, appendicectomy has been the gold standard treatment for acute appendicitis. This requires an inpatient stay, a surgical operation requiring general anaesthesia, and exposure to potential complications not only of the disease but also surgery. Whilst the majority of children recover from acute appendicitis, the disease carries a mortality of 0.08-0.31 per 1,000 cases in children [2].

In recent years, there has been growing interest in alternatives to appendicectomy. In particular, non-operative treatment of appendicitis, with antibiotics alone, has been proposed as a potential treatment. A small number of randomised controlled trials (RCTs) in adults [3–5] and, more recently, children [6, 7] suggest that antibiotic treatment may be a valid alternative to appendicectomy. However there is currently insufficient data to justify its widespread use. Prior to performing further efficacy studies of the treatment of appendicitis in children, it is imperative to identify the most relevant outcome measures for inclusion in the design of comparative studies. This is of particular importance when evaluating a novel treatment approach since the outcomes of importance may differ from those commonly reported with traditional therapies.

Core outcome sets (COS) have been proposed as a means of standardizing outcome selection, measurement and reporting in healthcare research and in clinical trials in particular [8, 9]. The development of a COS and its adoption by researchers is intended to help avoid inconsistencies in outcome selection, measurement and reporting that may otherwise exist. The lack of a COS may result in (i) important outcomes being overlooked or omitted in study design, (ii) inconsistent definitions or measurement techniques being used across studies and, (iii) omission of important outcomes from reports (reporting bias) [8]. Selecting outcomes that are important to a range of stakeholders is important if research is to be meaningful and relevant. If trials do not adopt an established COS they risk selecting suboptimal outcomes and are unlikely to contribute usable information [10]. The use of outcomes within a COS should also improve evidence synthesis across multiple studies (for example, meta-analysis) by removing inconsistencies in outcome selection, definition and reporting.

A review of the relevant literature and electronic resources failed to identify a COS for children with appendicitis. As the first stage of a process to develop a COS, we designed a study to determine which outcomes are currently reported in RCTs and systematic reviews (SRs) investigating treatment interventions in children (≤18 years) with acute appendicitis.

## Methods

This review was performed in accordance with the PRISMA guidelines for systematic reviews (see Additional file 1 for compliance details) [11] and according to a defined protocol (provided as Additional file 2).

### Study selection criteria

Studies from the existing literature were selected in order to address our specific research question using the following criteria.

#### Types of studies

We included systematic reviews of RCTs with or without meta-analysis, and randomised controlled trials.

#### Types of intervention

Any medical or surgical intervention intended as treatment, or as a component of the treatment, of patients ≤18 years of age (that is, children, infants or babies) with acute appendicitis was included. For the purposes of this review, we did not apply a specific definition of acute appendicitis, for example, one based on diagnostic imaging or pathology findings, because not all participants of individual studies had diagnostic imaging or pathology on a surgical specimen. This is consistent with contemporary surgical practice. Rather, we included studies on the basis that the population being reported had a diagnosis of acute appendicitis, regardless of how it was defined in the individual study.

#### Types of participants

Participants were children aged ≤18 years with acute appendicitis.

#### Exclusion criteria

Studies in which the purpose of the intervention was for symptom control rather than treatment of disease including analgesic interventions and interventions to treat nausea and vomiting, studies comparing one or more diagnostic techniques, RCTs that reported a population that included any patient over 18 years of age without a subgroup analysis containing only children ≤18 years, studies that included any patient with a diagnosis other than acute appendicitis, studies reported in abstract form only such as conference proceedings, and any study that was not written in English were all excluded. Interim reports of a study for which the final report was included were excluded as were prior versions of SRs for which the review had been more recently updated and was included.

##### Search strategy

Searches were conducted by an academic health information specialist (CN) in April 2014 in the following databases: MEDLINE (1946 - 22 April 22 2014 and including the “In-process & Other Non-Indexed” segment), Embase (1947 to 2014 Week 16) and Cochrane Central Register of Controlled Trials - CCRCT (1991 - 22 April 2014). Database specific subject headings were selected for the concepts of appendicitis, children and randomised controlled trial study design. Database subject headings were exploded, when applicable, to include narrower terms. Free text word searches were generated for all of the concepts using the database ‘Used For’ terms. In all databases, adjacency operators and truncation symbols were used in text word searches when appropriate to capture variations in phrasing and expression of terms. All synonymous terms were combined first using the Boolean “OR.” The three distinct concepts related to intervention, population, and study design were combined with the Boolean “AND” in MEDLINE and Embase. In CCRCT, only the concepts of appendicitis and children were searched and combined with “AND.” No language or date restrictions were applied. A detailed search strategy for MEDLINE is described in Appendix 1. We limited our search to the three electronic databases and did not search other sources with the exception of searching the reference lists of SRs for RCTs that were not identified by our literature search.

### Study selection

Two reviewers (NJH and MZK) independently assessed the titles and abstracts of all identified citations. Full-text articles were retrieved if either reviewer considered the citation potentially relevant with a low threshold for retrieval. Full texts of selected studies were then critically reviewed to assess eligibility. Reasons for exclusion of studies were recorded. The bibliographies of studies included for full-text review were also evaluated for additional relevant references. The final set of studies which was included in the systematic review was determined by consensus (between NH and MZK) with any disagreements resolved by a third reviewer (SE).

### Data extraction

Data were extracted independently and in duplicate by two reviewers (NJH and MZK) who then reviewed the extracted data together to ensure accuracy. Disagreements were resolved by a third reviewer (SE) when necessary. The following data were extracted from each study: study design (RCT or SR), year of study, region of origin, sample size, use of a primary outcome, all outcomes reported, and provision of a definition of each outcome. An outcome was included as reported whether it was included in the methods section, results section, or both. A study was deemed to use a primary outcome if the words ‘primary outcome’ were stated in the report, if data for a particular outcome were used to generate a sample size for a study, or if the stated aim of a study was to investigate the effect of an intervention on a single specific outcome or single defined composite outcome.

### Assessment of the similarity of outcomes

We anticipated diversity in the terminology used to report outcomes and therefore grouped similar outcomes. We identified outcomes that seemed similar or of a similar theme despite differing definitions used across studies and assigned an appropriate term to them. For instance, the outcomes ‘fever on post-operative day 3’, ‘duration of post-operative fever’ and ‘episodes of fever’ were all included in the term ‘post-operative fever’. We defined an additional outcome term of ‘other single outcome’ when an outcome was reported by a single study that could not naturally be mapped to any other outcome term.

Where a composite outcome was used we considered each individual component of the composite individually and included each in analysis but excluded the composite outcome from analysis. For example the outcome ‘surgical site infection’ was excluded unless its components of ‘wound infection’ and ‘intra-abdominal abscess’ were not reported separately.

### Assignment of outcome terms to core areas

Each individual outcome term was assigned to one of five core areas identified from the OMERACT Filter 2.0 [12]. The OMERACT Filter 2.0 is a framework developed to ensure that a full breadth of outcomes is reported by RCTs during the development of a COS. The OMERACT Filter 2.0 includes the four core areas of ‘Death’, ‘Pathophysiological Manifestations’, ‘Life Impact’, and ‘Resource Use’. It further recommends that ‘Adverse Events’ are reported across all core areas. For the purposes of this review, we included ‘Adverse Events’ as a fifth core area. Terms were mapped to core areas independently by two reviewers (NJH and MZK), and discrepancies were resolved by a third reviewer (SE) as necessary. We then determined the number of studies that provided an outcome within each core area and the number of core areas covered by each individual study in this review.

### Data synthesis

The total number of studies identified and included and the number of different outcomes identified in both RCTs and SRs were counted and reported separately to avoid double counting of outcomes. Although some of the RCTs do contribute to some of the SRs, our descriptive reporting means that this does not impact our findings. The number of outcome terms, variations in definition for each outcome term and number of terms reported by each study are reported and illustrated graphically. Finally, we identified the number of outcome terms assigned to each core area and the number of core areas covered by each included study. All these data are reported descriptively with appropriate summary measures for non-parametric data. Since we did not capture quantitative outcome data from individual studies, rather which outcomes were selected and reported, it is inappropriate to assess heterogeneity between studies formally using an I^2^ statistic or similar.

## Results

Identification and description of included studies.

**Fig. 1:**
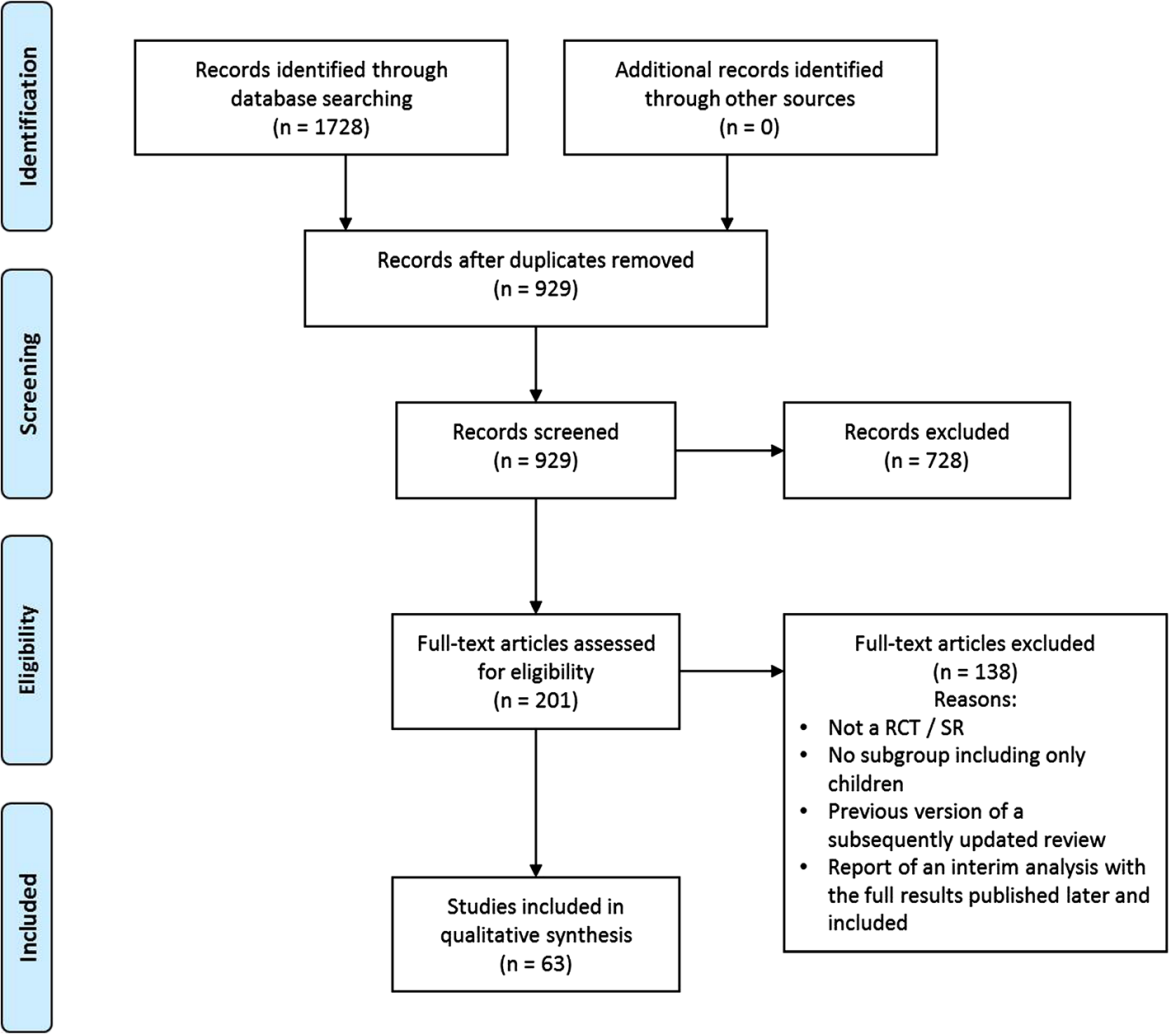
PRISMA article selection flow diagram

A flow diagram detailing article selection is shown in Fig. 1. Our search yielded a total of 1,728 articles (620 from Medline, 633 from EMBASE, and 475 from CCRCT). Screening of 929 non-duplicate titles and abstracts identified 201 citations to undergo full text review. One study [13] was excluded as it reported interim results of a trial that was subsequently reported in full and included in this review [14]. Early versions of two SRs were excluded [15–18] as they were prior versions of subsequently updated (and included) SRs [19, 20].

A total of 63 articles met the specified inclusion criteria and were included in this review. Nine of these were SRs, with or without meta-analysis, including two Cochrane reviews [19, 20]. The remaining 54 published articles reported outcomes from 51 RCTs, as three of the RCTs provided data for two articles each. Myers *et al*. [21] reported cost-related outcomes of the trial for which clinical outcomes were separately reported by Blakely *et al*. [22]. Schurman *et al*. [23] reported patient and parent quality of life outcomes of the trial for which clinical outcomes were reported by St Peter *et al*. [24]. Gasior *et al*. [25] reported cosmetic outcomes of the trial for which the clinical outcomes were reported by St Peter *et al*. [26]. For the purposes of analysis, the data arising from these three supplementary articles were amalgamated with the clinical outcomes. We therefore present outcomes reported in 51 RCTs and 9 SRs.

The characteristics of included studies are found in Table 1. Years of publication were between 1973 and 2013 for RCTs and between 2005 and 2013 for SRs. The median total sample size for each RCT was 100 children (range 25 to 1083). The region of origin for the RCTs included North America (n = 18), Europe (n = 22), Asia (n = 8), Africa (n = 1), Australasia (n = 2) and Central America (n = 1). The interventions investigated in each study fell into two broad categories: (A) investigation of antibiotic type, route of administration, or duration; (B) investigation of a type of surgical intervention, including type and timing of wound closure, use of a peritoneal drain, type of peritoneal access (for example, open, laparoscopic, single incision laparoscopic), timing of appendicectomy, and type of insufflation gas during laparoscopic appendectomy. A total of 28 RCTs fell into category A and 23 into category B. Two systematic reviews were related to antibiotic use (category A) and seven to type of surgical intervention (category B).

**Table 1:** Characteristics of included studies

First Author	Year	Intervention type	Study type	Region of origin	Sample size	Age of included population
Haller Jr, [37]	1973	Surgical	RCT	NA	43	0 to 14 years
Bates [38]	1974	Antibiotic	RCT	Europe	38	0 to 9 years
Fowler [39]	1975	Antibiotic	RCT	Australasia	69	‘children’
Sherman [40]	1976	Surgical	RCT	NA	79	13 month to 16 years
Giacomantonio [41]	1982	Antibiotic	RCT	NA	42	‘pediatric age range’
Hutchinson [42]	1983	Antibiotic	RCT	Europe	133	16 month to 15 years
King [43]	1983	Antibiotic	RCT	NA	64	<18 years
Foster [14]	1987	Antibiotic	RCT	Europe	100	5 to 14 years
Gutierrez [44]	1987	Antibiotic	RCT	Europe	100	<10 years
Thomson [45]	1987	Antibiotic	RCT	Europe	84	4 to 13 years
McAllister [46]	1988	Antibiotic	RCT	Europe	401	‘children’
Schmitt [47]	1989	Antibiotic	RCT	Europe	64	6 months to 15 years
Kooi [48]	1990	Antibiotic	RCT	Asia	100	<13 years
Meller [49]	1991	Antibiotic	RCT	NA	59	2 to 15 years
Pokorny [50]	1991	Antibiotic	RCT	NA	95	2 to 12 years
Schropp [51]	1991	Antibiotic	RCT	NA	97	<18 years
Kizilcan [52]	1992	Antibiotic	RCT	Europe	100	0 to 15 years
Tsang [53]	1992	Surgical	RCT	Asia	63	2 to 12 years
Uhari [54]	1992	Antibiotic	RCT	Europe	218	2.5 to 16.8 years
Banani [55]	1995	Antibiotic	RCT	Asia	246	4 to 15 years
Soderquist-Elinder [56]	1995	Antibiotic	RCT	Europe	544	‘children’
Toki [57]	1995	Surgical	RCT	Asia	53	2 to 14 years
Lejus [27]	1996	Surgical	RCT	Europe	63	8 to 15 years
Ciftci [58]	1997	Antibiotic	RCT	Europe	200	1 to 16 years
Banani [59]	1999	Antibiotic	RCT	Asia	1083	4 to 15 years
Gorecki [60]	2001	Antibiotic	RCT	Europe	152	‘children’
Lavonius [61]	2001	Surgical	RCT	Europe	43	7 to 15 years
Lintula [62]	2001	Surgical	RCT	Europe	61	4 to 15 years
Rice [63]	2001	Antibiotic	RCT	NA	26	5 to 18 years
Shalaby [64]	2001	Surgical	RCT	Africa	150	7 to 14 years
Lintula [65]	2002	Surgical	RCT	Europe	25	4 to 15 years
Little [66]	2002	Surgical	RCT	NA	129	1 to 16 years
Tander [67]	2003	Surgical	RCT	Europe	140	Mean 7.1 year
Lintula [68]	2004	Surgical	RCT	Europe	87	4 to 15 years
Oka [69]	2004	Surgical	RCT	NA	517	Mean 10.7 years
Snelling [70]	2004	Antibiotic	SR	NA	2284	<21 years
Andersen [19]	2005	Antibiotic	SR	NA	NR	‘children’
Henry [71]	2005	Surgical	SR	NA	107	‘children’
Schneider [72]	2005	Antibiotic	RCT	Europe	27	Mean 10.3 years
Aziz [73]	2006	Surgical	SR	NA	6,477	‘children’
Malik [34]	2007	Surgical	RCT	Asia	120	3 to 18 years
Padankatti [74]	2008	Surgical	RCT	Asia	30	2 to 14 years
St Peter [1]	2008	Antibiotic	RCT	NA	100	Mean 8.6 years
Fraser [75]	2010	Antibiotic	RCT	NA	102	Mean 9.9 years
Pauniaho [76]	2010	Surgical	RCT	Europe	198	4 to 18 years
Saha [77]	2010	Surgical	RCT	Asia	60	<12 years
Sauerland [20]	2010	Surgical	SR	NA	542	‘children’
St Peter[24]	2010	Surgical	RCT	NA	40	<18 years
Blakely [22]	2011	Surgical	RCT	NA	131	<18 years
Perez [78]	2011	Antibiotic	RCT	CA	100	2 to 12 years
Romero [35]	2011	Antibiotic	RCT	Europe	49	5 to 15 years
Schurman [23]	2011	Surgical	RCT	NA	40	<18 years
St Peter [26]	2011	Surgical	RCT	NA	360	<18 years
Esposito [79]	2012	Surgical	SR	NA	123,628	0 to 18 years
Markar [80]	2012	Surgical	SR	Europe	107,624	‘children’
Myers [21]	2012	Surgical	RCT	NA	131	<18 years
St Peter [81]	2012	Surgical	RCT	NA	220	<18 years
Yu [82]	2013	Surgical	RCT	Australasia	190	8 to 14 years
Dalgic [83]	2013	Antibiotic	RCT	Europe	107	3 months to 17 years
Gasior [25]	2013	Surgical	RCT	NA	198	<18 years
Li [84]	2013	Surgical	SR	Asia	873	children >1 year
Nataraja [85]	2013	Surgical	SR	Europe	22,060	<18 years
Perez [86]	2013	Surgical	RCT	NA	50	2.9 to 15.7 years

*CA* Central America, *NA* North America, *NR* not reported, *RCT* randomised controlled trial, *SR* systematic review

### Description of outcomes in RCTs

**Fig. 2:**
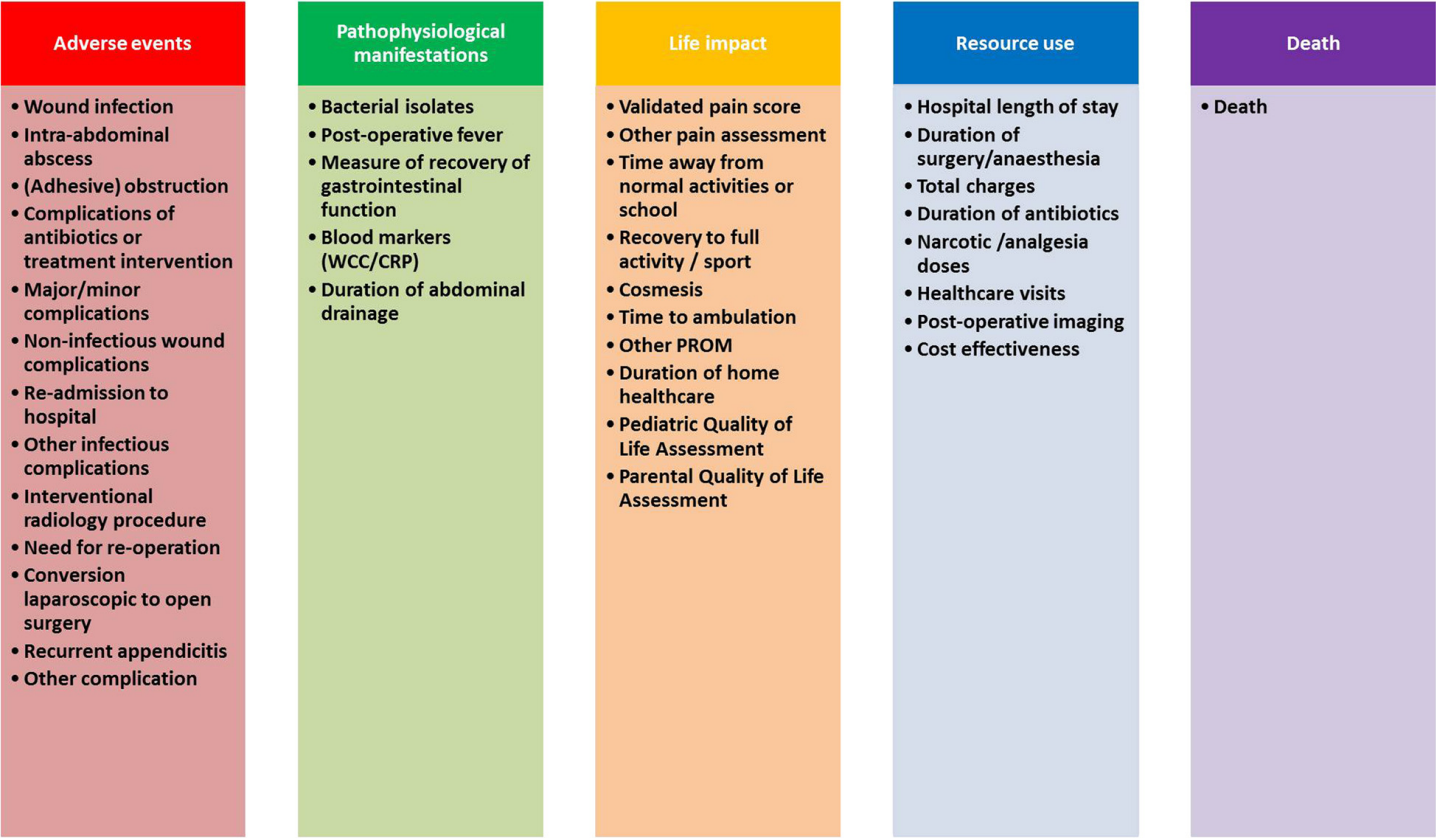
Assignment of outcome terms to core areas. WCC white cell count, CRP C-reactive protein, PROM patient reported outcome measure

**Fig. 3:**
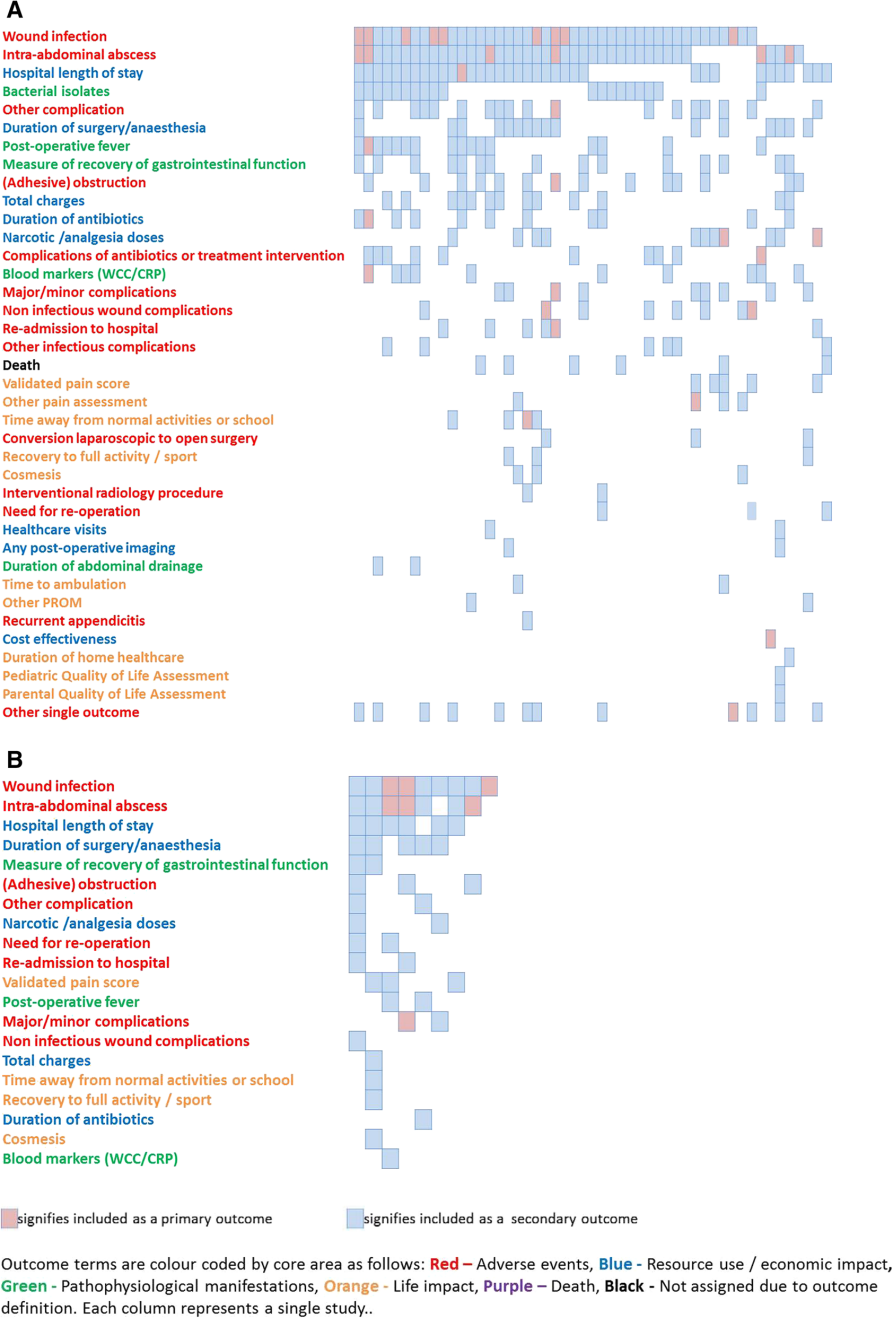
**a** Outcome matrix of 38 outcome terms for 51 RCTs **b**. Outcome matrix of 20 outcome terms reported in 9 systematic reviews (SRs)

A total of 115 different unique outcomes were identified in the 51 RCTs. One hundred and six of these outcomes were mapped to the 38 *terms* shown in Fig. 2, and 12 outcomes were mapped to the additional term ‘other single outcome’. Each RCT reported a median of nine “unmapped” outcomes (range 1-14). The most frequently reported outcome was wound infection, which was reported in 43 RCTs (84.3 %) and was the primary outcome or a component of a composite primary outcome in nine RCTs (17.6 %). Figure 3a shows the distribution of primary and secondary outcome terms across individual RCTs. A primary outcome was identified in 26 RCTs (50.9 %). In addition to a wide range in number of outcomes reported in each study, there was great heterogeneity in terms reported across studies. For example only 25 of the 51 (49 %) RCTs reported all of the three most frequently reported terms (wound infection, abscess formation and length of stay). Definitions are shown in Table 2 for outcomes identified in RCTs for which there may be variability dependent on the definition. We identified multiple definitions used for many outcomes, apparently leading to different occurrence frequencies of “similar outcomes” across studies. For wound infection, the most frequently reported outcome, there were a total of 11 different definitions identified in 43 RCTs, 31 of which reported no definition. Finally, work was undertaken to ascertain whether any substantial changes occurred in the reported outcomes over the period of publication covered by this study (1973-2013), but none was found.

**Table 2:** Variation in definition of outcomes in randomised controlled trials

Definition of wound infection (n = 43)	
	Presence of pus in the wound or wound pain, tenderness or erythema of sufficient magnitude to interfere with the patients well-being or to prolong hospital stay or to require readmission for wound infection [41]
	Discharge of pus from the wound [14, 42, 47]
	Purulent material which drained either spontaneously or by surgical incision [43]
	Pus or erythema in the wound [49]
	Presence of gross purulent discharge with or without positive bacterial culture [53]
	Purulent discharge or positive culture from wound [55]
	Suppuration confirmed by spontaneous wound rupture, debridement or incision [56]
	Peri-incisional cellulitis or seropurulent wound drainage, whether culture positive or not [60]
	Local signs of inflammation plus positive bacterial culture [65]
	Clinical wound evaluation score on post-operative day 1, 2 and 7 [76]
	No formal definition (n = 31)
Definition of intra-abdominal/peritoneal abscess (n = 42)	
	Clinical symptoms plus laboratory findings of inflammation plus a positive ultrasound examination [65]
	Abscess within the abdominal cavity diagnosed at operation or by rectal examination, x-ray or ultrasound [56]
	Collections of purulent material which drained either spontaneously or by surgical incision [43]
	Deep abscess [42]
	No formal definition (n = 38)
Definition of post-operative fever (n = 16)	
	Duration of fever (maximum daily rectal temperature >101 °F) [43]
	Duration of fever [47]
	Incidence of post-op fever (rectal >101 °F) during first 3 post-operative days [49]
	Duration of fever (>100 °F) [50]
	Duration of fever (>101 °F) [51]
	Mean daily temperature; incidence fever (>37.5 °C) on third post-operative day [54]
	Duration of fever (>38.0 °C) [55]
	Duration of fever (>37.0 °C) [57, 58]
	Temperature on first post-operative day [60]
	Incidence of persistent fever (>38.5 °C for >3 days) [63]
	Incidence of post-operative fever [66]
	Maximum daily temperature for first five post-operative days [1, 75, 83]
	Temperature on day 4 of treatment; percentage afebrile by day 7 of treatment [78]
Definition of post-operative leucocytsis (n = 8)	
	Duration of white cell count (WCC) >12,500/cm^3^ [43]
	Pre-discharge WCC [51]
	WCC on days 4 and 8 of treatment [78]
	Duration of WCC >10 x 10^9^/L [58]
	Resolution of leukocytosis [60]
	Time to return to normal WCC [63]
	Trend of WCC reduction [55]
	WCC on post-operative days 1 and 2 [76]
Definition of time away from normal activity (n = 4)	
	Return to school [40, 65]
	Resumption of normal activity [66]
	Time away from normal activities (a combination of objective time periods (hospital length of stay, outpatient status with central venous catheter, and receiving intravenous antibiotics) and more subjective time periods (for example, outpatient with symptoms that limit activity)) [22]
Definition of time away from full activity (n = 3)	
	Able to do full physical activity at 1 and 4 weeks [61]
	Return to sport activities [65]
	Days to full activity [26]
Definition of cosmesis (n = 3)	
	Visual cosmesis score on eighth post-operative day [34]
	Parental dissatisfaction with cosmetic result at 10 and 90 days; assessment by surgeon (mean of two blinded assessors) using a visual analogue scale at 90 days [35]
	Patient scar assessment questionnaire (validated in adults) at 6 weeks and 18 months [25]

### Description of outcomes in systematic reviews

A total of 31 different outcomes were identified in the nine SRs and were mapped to the same terms shown in Fig. 2. The most frequently reported outcome in SRs was also wound infection, which was reported in all SRs. The distribution of primary and secondary outcome terms across individual SRs is shown in Fig. 3b. A primary outcome was identified in four SRs, including a composite primary outcome in two. Definitions of outcomes used in SRs are shown in Table 3. Outcomes were generally poorly defined in SRs, which often replicated the definition used in individual studies contributing to the review.

**Table 3:** Variation in definition of outcomes in systematic reviews

Definition of wound infection (n = 9)	
	Discharge of pus from the wound [19, 71]
	Wound infection within the first month of surgery as a direct result of the initial operation [80]
	No formal definition (n = 6)
Definition of intra-abdominal abscess (n = 7)	
	Postoperative intra-abdominal abscess (persistent pyrexia without any other focus, after operation, palpable mass in the abdomen or discharge of pus from the rectum) [19]
	No formal definition (n = 6)
Definition of post-operative fever (n = 2)	
	Duration of fever [70]
	Post-operative fever [73]
Definition of leucocytosis (n = 1)	
	Duration of leukocytosis [70]
Definition of time to normal activity (n = 1)	
	Return to normal activity [20]
Definition of time to full activity (n = 1)	
	Return to full activity; return tosports [20]
Definition of cosmesis (n = 1)	
	Cosmesis measured on visual analogue scale [20]

### Assignment of outcome terms to core areas

Thirty-seven of the 38 outcome terms were assigned to core areas defined by OMERACT Filter 2.0; the outcome term ‘other single outcome’ could not be mapped due to its definition and inherent heterogeneity. The assignment of outcome terms to core areas is shown in Fig. 2. The core area populated most heavily was ‘Adverse Events’ (n = 13 outcome terms), followed by ‘Life Impact’ (n = 10 outcome terms). With the exception of the core area ‘Death’, the core area of ‘Pathophysiological Manifestations’ was populated least (n = 5 outcome terms).

**Fig. 4:**
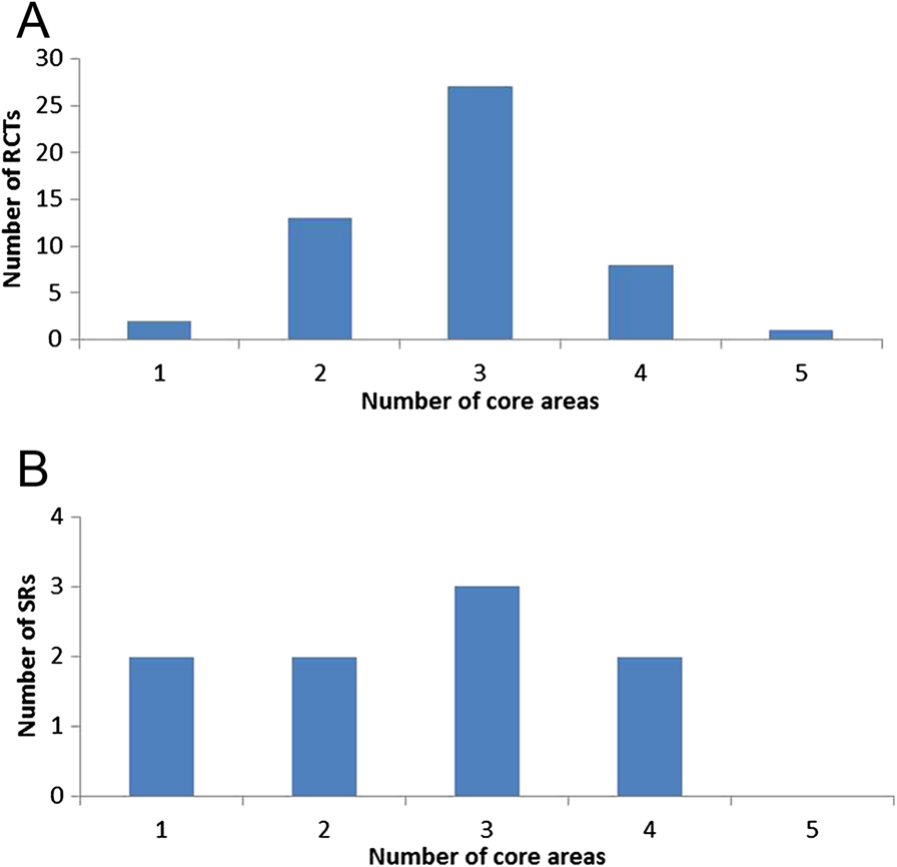
**a** Distribution of core areas to which outcome terms were assigned in RCTs. **b** Distribution of core areas to which outcome terms were assigned in SRs

All RCTs and SRs reported at least one outcome assigned to the ‘Adverse Event’ core area. The median number of core areas to which outcome terms were assigned in both RCTs and SRs was three. Two RCTs reported outcomes assigned to just one core area, and only one RCT [27] reported outcomes assigned to all five core areas (Fig. 4a). For SRs, two studies reported outcomes in just one core area, whereas no SR reported outcomes in all five core areas (Fig. 4b). With the exception of death (reported in 6 RCTs and no SRs) the core area ‘Life Impact’ was reported least frequently (15 RCTs and 3 SRs).

## Discussion

We have analysed outcome reporting from a large number of RCTs and SRs relating to the treatment of acute appendicitis in children. The principal findings of our review are 1) a wide heterogeneity of outcomes reported between individual studies, 2) the lack of a standardised definition for commonly reported outcomes (including absence of any definition), 3) little overlap in outcomes used across studies, and 4) a relative paucity of studies reporting patient relevant outcomes within the ‘Life Impact’ core area. These findings have implications for the validity of comparisons made between individual RCTs, not only within meta-analyses, but also for the practising surgeon when making a best practice judgement based on best available evidence. Our findings also suggest that patient or parent involvement in defining appropriate outcomes for this condition has been limited. The majority of outcomes are highly clinically focused, suggesting that they have been proposed by and considered important to, clinicians. A similar phenomenon was identified by a recent review of studies reporting COS development [10]. Overall, our study provides justification for the development of a COS for acute appendicitis in children. It also highlights the need for such an outcome set to use standardised definitions of included outcomes and for the involvement of multiple key stakeholder groups, particularly patients and parents, in both identifying candidate outcomes and within any consensus process leading to definition of a COS. In line with the principles of COS development, such a COS for paediatric appendicitis has the capacity to improve the quality of outcome selection and reporting carrying clear benefits for researchers, clinicians and patients.

This study suggests that the outcomes judged to be of greatest relevance to clinicians and researchers are wound infection and intra-abdominal abscess formation. At least one of these outcomes was reported in 94 % of RCTs and all SRs. Despite this, only a minority of each type of study used a standardised definition for either outcome. A wide variety of definitions were also used for other frequently reported outcomes. The issue of a lack of definition of commonly reported outcomes is not unique to paediatric appendicitis. Previous studies of other health conditions have highlighted variability in definitions [28–30] including one study that identified 56 separate definitions for the same outcome across a review of 97 studies [31]. The lack of definition and variation in definitions across a single outcome severely hampers attempts to compare results between studies. A precise and standardised definition of any outcome measure is one of the key principles underlying selection of an outcome within a COS.

A large number of studies (nearly half of all RCTs and over half of SRs) did not define a primary outcome. It is therefore not possible to determine which outcome researchers considered most important to measure for these studies. Further consequences of the lack of primary outcome are the possibility of reporting bias since it is not known whether only statistically significant outcomes are reported and that these studies may have lacked sufficient power to determine effect of treatment intervention.

We identified further methodological inconsistencies that may also act as a source of bias within RCTs. For instance, a number of RCTs stated that an outcome would be measured in the methods section but it was then never actually reported. Whilst we do not believe this type of inconsistency has impacted our results because we specifically included outcomes mentioned in either ‘methods’ or ‘results’ section of each report, this observation clearly reflect reporting bias within individual studies. Other methodological issues encountered within RCTs included randomization techniques that may be subject to lack of concealment. Whilst some reviews of outcome reporting have included an assessment of the methodological quality of RCTs to identify potential sources of bias [29], we did not complete a full methodological assessment for each study included in our review. We do not believe that other biases that may exist within RCTs have influenced the primary objective of our study, namely to identify outcomes reported by existing RCTs and SRs. The exception to this is reporting bias for which we used a specific strategy to identify outcomes that were planned to be reported as well as those that actually were. As a result we feel the chances that we have missed important outcomes are minimal.

In recent years, the COMET (Core Outcome Measures in Effectiveness Trials) initiative [32] has strongly supported the development of core outcome sets to standardise outcome definitions and measurement for studies that assess the efficacy of a treatment. The expectation is that a trial investigating treatment of a condition should always measure and report (as a minimum) each outcome within a COS where one exists for that condition. Further guidance from groups such as OMERACT [12, 33] has supported the importance of reporting a wide breadth of outcomes across a number of core areas to ensure that each COS is relevant for multiple stakeholder groups and in particular the patient. Whilst all five core areas were fulfilled with outcomes in our review, outcomes which may be of greater relevance to patients or parents, or provide an assessment of outcome from a patient or parental perspective in the ‘Life Impact’ core area were rarely reported. For example, only three of the 23 RCTs relating to type of surgical intervention reported any marker of cosmesis as an outcome [26, 34, 35], and only one RCT used a validated patient or parental quality of life assessment tool [23]. Based on our experience in this review, the OMERACT filter 2.0 likely acts as a useful framework for researchers to ensure that all core areas are fulfilled when developing a COS and designing a RCT.

However, the OMERACT Filter 2.0 may have some limitations. It was developed primarily for designing trials within the field of rheumatology [12] and has subsequently been proposed as an appropriate framework for other fields [33]. Hence there may be specialty-specific or age-group specific factors that affect the suitability of the filter for other fields. In assigning outcome terms to OMERACT core areas, we encountered several examples where an outcome term could potentially be assigned to more than one core area. For example, hospital length of stay could be considered in the context of (ongoing) ‘Pathophysiological Manifestations’, or within ‘Resource Use’ or ‘Life Impact’. We did not identify any outcome terms that could not be readily assigned to any of the OMERACT core areas but would urge those developing core outcome sets to be critical in their assignment of outcomes to ensure that all core areas are genuinely covered in any COS.

In addition to proposing core areas, the OMERACT framework goes further, identifying essential characteristics of outcome measures within a COS which should be ‘truthful’, ‘discriminative’ and ‘feasible’ [12]. The lack of definitions of outcomes we have identified would clearly not pass these criteria. It is essential that this is addressed in the development of a COS. Whilst it is possible that the reason important core areas such as ‘Life Impact’ have been relatively ignored in existing studies is because of a lack of reliable and valid measures, we suspect this it is not the case and that a focus on clinician relevant outcomes is a more likely explanation.

The principal strengths of this systematic review are the extensive literature searches in multiple bibliographical databases over a long time period. We captured a wide range of outcomes with a variety of definitions. Whilst a limitation of our methodology to include only RCTs and SRs means that it is possible that other outcomes reported in other types of study have been missed, we are confident that our methodology has enabled us to capture outcomes that researchers and clinicians consider important. The main weakness of our study is that our search for outcomes has been limited to those reported in the existing literature. We are unable to comment on outcomes that may not have been reported in the existing literature or are important to other stakeholder groups, in particular patient and parents, but also other health professionals such as nurses and family doctors. The importance of engaging patients and parents in research and in particular in defining outcomes of importance is being increasingly recognized. Only by ensuring that patients and parents are involved in determining which outcomes should be measured can we be confident that treatment interventions are investigated in a way that is relevant to the target population. This aspect will be key in developing a COS. Outcomes may also not have been reported in RCTs and SRs due to selective reporting bias, a relatively common phenomenon [36]. While our systematic review highlighted the heterogeneity of outcome reporting, outcome reporting bias has a more detrimental effect on quantitative meta-analyses to establish benefit (or harm) of an intervention, which was not the objective of our current study.

Although acute appendicitis is the most common abdominal surgical emergency in children, and is one of the few areas in paediatric surgery that has been the subject of multiple RCTs, there is no COS for the condition. Existing outcomes are heavily biased towards clinician and researcher areas of interest rather than patient/parent relevant factors and do not use standardised definitions. This study supports our commitment to develop a COS for acute appendicitis in children to ensure that outcomes measured in future studies of existing and novel therapies are relevant to multiple stakeholder groups and that studies can be compared, combined and contrasted meaningfully. We now intend to complete a consensus process amongst these stakeholder groups to develop a COS for acute appendicitis in children consistent with the principles of the OMERACT framework and for use in future trials of treatment interventions.

## Conclusions

There is a wide heterogeneity in the selection and definition of outcomes in RCTs and SRs of paediatric appendicitis with little overlap in outcomes used across studies. A paucity of studies report patient relevant outcomes within the ‘Life Impact’ core area. These factors impair meaningful evidence synthesis, and pose challenges to those designing prospective clinical trials and cohort studies. The commitment to develop a COS for paediatric appendicitis is justified.

## Appendix 1

**Search strategy for MEDLINE**

Database: Ovid MEDLINE(R) 1946 to Present with Daily Update, Ovid MEDLINE(R) In-Process & Other Non-Indexed Citations <April 21, 2014>

Search Strategy:

1. Appendicitis/ (15193)
2. Appendix/ (5012)
3. Appendectomy/ (8492)
4. (appendix or appendicitis or appendicectom* or appendectom* or "vermiform process*" or "processus vermiformis").mp. (31995)
5. or/1-4 (31995)
6. exp Randomized Controlled Trials as Topic/ (93075)
7. exp randomized controlled trial/ (371186)
8. meta-analysis/ (47125)
9. meta-analysis as topic/ (13675)
10. cross-over studies/ or double-blind method/ or random allocation/ or single-blind method/ (232109)
11. (random* or rct*).mp. (891252)
12. (pragmatic adj2 trial*).mp. (735)
13. ("meta-analys*" or metaanalys*).mp. (82887)
14. "systematic review*".mp. (52825)
15. (medline or embase or lilacs or wos or "web of science" or scopus or cochrane).ti,ab. (72530)
16. ((doubl* or singl* or tripl*) adj2 (blind* or mask*)).mp. (178525)
17. ((cross-over or crossover) adj2 (design* or stud*or trial*)).mp. (53183)
18. or/6-17 (1028267)
19. 5 and 18 (1365)
20. limit 19 to "all child (0 to 18 years)" (555)
21. (infan* or newborn* or new-born* or neonat* or baby or babies or child* or youth or kid or kids or toddler* or boy* or girl* or adolescen* or teen* or juvenile* or p?ediatric*).mp. (3381275)
22. 19 and 21 (620)
23. 20 or 22 (620)

## Additional files

Additional file 1:
**PRISMA 2009 Checklist.**
Additional file 2:
**Protocol.**

## Abbreviations

COMET: Core Outcome Measures in Effectiveness Trials
COS: core outcome set
CRP: C-reactive protein
OMERACT: outcome measures in rheumatology clinical trials
PROM: patient reported outcome measure
RCT: randomised controlled trial
SR: systematic review
WCC: white cell count
