# Supplementary material for: Outcome reporting in randomised controlled trials and meta-analyses of appendicitis treatments in children: a systematic review
Source: Trials. 2015 Jun 17;16:275. doi: 10.1186/s13063-015-0783-1 (PMC4499220; doi:10.1186/s13063-015-0783-1)
Supplement: Additional file 2: — Protocol. [file 13063_2015_783_MOESM2_ESM.pdf]

Outcome reporting in randomised controlled trials and meta-analyses of appendicitis treatments in children: a systematic review

Review Team
Nigel J Hall, Mufiza Kapadia, Simon Eaton, Winnie WY Chan, Cheri Nickel, Agostino Pierro, Martin Offringa

Review question(s)
Primary aim is to identify all outcomes measured in randomised controlled trials (RCTs) or systematic reviews (SRs) of any treatment intervention (and any comparator) in children with acute appendicitis.

Searches
Searches within: MEDLINE (including the "In-process & Other Non-Indexed" segment), Embase Cochrane Central Register of Controlled Trials - CCRCT. See Appendix 1 for full search strategies. Search all databases from inception until date of search.

Types of study to be included
Randomised controlled trials (RCTs) and Systematic reviews (SRs) of treatment interventions for acute appendicitis in children

Condition being studied
Acute appendicitis

Participants/ population
Children

Intervention(s)
Any

Comparator(s)
Any

Outcome(s)
Primary outcomes (both reported and intended to be reported)
All outcomes (both reported and intended to be reported)

Data extraction
Each abstract will be independently read by two authors (one methodologist and one clinician), for deciding whether full-text should be retrieved. If at least one will suggest retrieval, full-text will be retrieved.
Two authors (one methodologist and one clinician) for final check on eligibility will read each retrieved full-text. In case of disagreement, a third opinion will be sought. Further uncertainties will be resolved by consensus.

The following information will be extracted for each study

1) Bibliographic details (authors, title, publication date)
2) Study type: RCT or SR

3) Region of origin
4) Type of intervention / comparator (antibiotic related or surgical intervention)
5) Sample size
6) Age of population
7) For each outcome in each study:
  i) type of outcome: primary vs secondary
  ii) outcome definition

Data extraction from each full-text will be performed independently by two authors; and discrepancies will be resolved by reviewing the full-text and by consensus.

Strategy for data synthesis
Descriptive statistics of all characteristics of the included RCTs and SRs will be produced. A list of all outcomes identified in the included studies will be provided.. Outcomes will be assessed for similarity and mapped to outcome terms. The number of studies reporting each outcome term will be reported. These outcome terms will then be classified in the following core areas from the OMERACT Filter 2.0: 'Death', 'Pathophysiological Manifestations', 'Life Impact', 'Resource Use' and 'Adverse Events'. This assessment will be made independently by a clinician and a methodologist; discrepancies will be resolved by consensus. The number of core areas to which an outcome term is assigned in each study will be determined.

Analysis of subgroups or subsets
1) results for RCTs and SRs will be presented separately to avoid double counting

Anticipated start date
1 April 2014

Anticipated completion date
31 December 2014

Funding sources/sponsors
Not applicable.

Language
English

Country
United Kingdom, Canada

Appendix 1: Search strategies for each database

1. MEDLINE

Database: Ovid MEDLINE(R) 1946 to Present with Daily Update, Ovid MEDLINE(R) In-Process & Other Non-Indexed Citations
Search Strategy:
----
1 Appendicitis/ (15193)
2 Appendix/ (5012)
3 Appendectomy/ (8492)
4 (appendix or appendicitis or appendicectom* or appendectom* or "vermiform process*" or "processus vermiformis").mp. (31995)
5 or/1-4 (31995)
6 exp Randomized Controlled Trials as Topic/ (93075)
7 exp randomized controlled trial/ (371186)
8 meta-analysis/ (47125)
9 meta-analysis as topic/ (13675)
10 cross-over studies/ or double-blind method/ or random allocation/ or single-blind method/ (232109)
11 (random* or rct*).mp. (891252)
12 (pragmatic adj2 trial*).mp. (735)
13 ("meta-analys*" or metaanalys*).mp. (82887)
14 "systematic review*".mp. (52825)
15 (medline or embase or lilacs or wos or "web of science" or scopus or cochrane).ti,ab. (72530)
16 ((doubl* or singl* or tripl*) adj2 (blind* or mask*)).mp. (178525)
17 ((cross-over or crossover) adj2 (design* or stud* or trial*)).mp. (53183)
18 or/6-17 (1028267)
19 5 and 18 (1365)
20 limit 19 to "all child (0 to 18 years)" (555)
21 (infan* or newborn* or new-born* or neonat* or baby or babies or child* or youth or kid or kids or toddler* or boy* or girl* or adolescen* or teen* or juvenile* or p?ediatric*).mp. (3381275)
22 19 and 21 (620)
23 20 or 22 (620)

2. EMBASE

Database: Embase Classic+Embase
Search Strategy:
----
1 acute appendicitis/ or appendicitis/ or appendix perforation/ (23611)
2 appendectomy/ (14628)
3 appendix/ (4768)
4 (appendix or appendicitis or appendicectom* or appendectom* or "vermiform process*" or "processus vermiformis").mp. (45936)
5 1 or 2 or 3 or 4 (45936)
6 randomized controlled trial/ or "randomized controlled trial (topic)"/ (390628)
7 meta analysis/ or "meta analysis (topic)"/ (88872)
8 crossover procedure/ or double blind procedure/ or single blind procedure/ or triple blind procedure/ (157081)
9 randomization/ (61907)
10 (random* or rct*).mp. (1018889)
11 (pragmatic adj2 trial*).mp. (856)
12 ("meta-analys*" or metaanalys*).mp. (118076)
13 "systematic review*".mp. (102090)
14 (medline or embase or lilacs or wos or "web of science" or scopus or cochrane).ti,ab. (85894)
15 ((doubl* or singl* or tripl*) adj2 (blind* or mask*)).mp. (209943)
16 ((cross-over or crossover) adj2 (design* or stud* or trial*)).mp. (49145)
17 or/6-16 (1223661)
18 5 and 17 (1993)
19 juvenile/ or exp adolescent/ or exp child/ (2857145)
20 (infan* or newborn* or new-born* or neonat* or baby or babies or child* or youth or kid or kids or toddler* or boy* or girl* or adolescen* or teen* or juvenile* or p?ediatric*).mp. (3610118)
21 19 or 20 (3610118)
22 18 and 21 (633)

3. CCRCT

Database: EBM Reviews - Cochrane Central Register of Controlled Trials
Search Strategy:
----
1 Appendicitis/ (311)
2 Appendix/ (27)
3 Appendectomy/ (352)
4 (appendix or appendicitis or appendicectom* or appendectom* or "vermiform process*" or "processus vermiformis").mp. (923)
5 or/1-4 (923)
6 (infan* or newborn* or new-born* or neonat* or baby or babies or child* or youth or kid or kids or toddler* or boy* or girl* or adolescen* or teen* or juvenile* or p?ediatric*).mp. (144104)
7 5 and 6 (475)
